# HIV-1 genetic diversity and primary drug resistance mutations before large-scale access to antiretroviral therapy, Republic of Congo

**DOI:** 10.1186/s13104-017-2550-8

**Published:** 2017-07-05

**Authors:** Fabien Roch Niama, Nicole Vidal, Halimatou Diop-Ndiaye, Etienne Nguimbi, Gabriel Ahombo, Philippe Diakabana, Édith Sophie Bayonne Kombo, Pembe Issamou Mayengue, Simon-Charles Kobawila, Henri Joseph Parra, Coumba Toure-Kane

**Affiliations:** 1 Laboratoire National de Santé Publique, Unité de Biologie Moléculaire, BP 120 Avenue du Général De Gaule, Brazzaville, Republic of Congo; 2 Laboratoire de Bactériologie et Virologie, Hôpital Le Dantec, Dakar, Sénégal; 3 Faculté des Sciences et Techniques, Université Marien Ngouabi, Brazzaville, Republic of Congo; 4 IRD_UMI 233 TransVIHMI, Délégation Régionale Occitanie 911 avenue Agropolis, Montpellier, France; 5 Faculté des Sciences de la Santé, Université Marien Ngouabi, Brazzaville, Republic of Congo

**Keywords:** HIV, Subtypes, Recombination, Drugs resistances, Congo

## Abstract

**Background:**

In this work, we investigated the genetic diversity of HIV-1 and the presence of mutations conferring antiretroviral drug resistance in 50 drug-naïve infected persons in the Republic of Congo (RoC). Samples were obtained before large-scale access to HAART in 2002 and 2004.

**Methods:**

To assess the HIV-1 genetic recombination, the sequencing of the *pol* gene encoding a protease and partial reverse transcriptase was performed and analyzed with updated references, including newly characterized CRFs. The assessment of drug resistance was conducted according to the WHO protocol.

**Results:**

Among the 50 samples analyzed for the *pol* gene, 50% were classified as intersubtype recombinants, charring complex structures inside the *pol* fragment. Five samples could not be classified (noted U). The most prevalent subtypes were G with 10 isolates and D with 11 isolates. One isolate of A, J, H, CRF05, CRF18 and CRF37 were also found. Two samples (4%) harboring the mutations M230L and Y181C associated with the TAMs M41L and T215Y, respectively, were found.

**Conclusion:**

This first study in the RoC, based on WHO classification, shows that the threshold of transmitted drug resistance before large-scale access to antiretroviral therapy is 4%.

## Background

A broad range of phylogenetic analyses of many isolates from various areas around the world revealed four groups of HIV-1: M, N, O and P [1]. Group M, which is more responsible for the global epidemic, is subdivided into subtypes (A–D, F–H, J and K), sub-subtypes (A1/A2/A3/A4, B/D, F1/F2), unique recombinant forms (URFs) and 71 circulating recombinant forms (CRFs) [2]. Several studies have indicated that the global distribution of HIV-1 subtypes is very heterogeneous, and some difference has been reported between continents and within countries, especially in central Africa, where the highest genetic subtype diversity has been found [3]. HIV diversity has been attributed to the error-prone nature of the reverse transcriptase (RT), which causes a high mutation rate and rapid turnover and generates frequent recombination between viral genomic RNA strands during their transcription into DNA [4, 5]. Furthermore, it was reported that the proportion of the recombinant virus CRFs and URFs are spreading and now play an important role in the global pandemic. Indeed, the prevalence of these viruses displaying designation of different subtypes in *gag* and *env* genes varies from less than 10 to 40%, depending on the country and population being studied [6]. In the neighbouring Congo countries, such as Cameroon, several subtypes of URFs and CRFs are co-circulating [2]. The CRF02_AG is more representative in many areas of the country and represents more than 65% of the isolates [7]. In the Democratic Republic of Congo (DRC), this rate changes between 32.9 and 59.3% [6, 7]. Previous reports showed the high genetic diversity concerning the distribution of HIV-1 subtypes in the Republic of Congo. The subtypes A, G and D predominate, but approximately 20–27% of strains circulating in the country are recombinants [7, 8] and 6.25% of strains remain unclassified.

Since 1986, in previously reported AIDS cases, the HIV epidemic has been rapidly spreading throughout the Republic of Congo (RoC) and becoming one of the most prevalent in west–central Africa. Based on a national second-generation survey performed in 2009, it was estimated that 3.2% of the population is infected. Approximately 30,000 people living with HIV/AIDS were in urgent need of antiretroviral therapy. To control the epidemic, the government of RoC has developed a national program to provide HAART (high antiretroviral therapy) since the end of 2003. Currently, approximately 21,000 people receive HAART, and 98% live in Brazzaville and Pointe-noire.

The present study aimed to contribute to the knowledge of the distribution of HIV-1 subtypes and recombinants in Congo-Brazzaville and to assess the presence of drug-resistance-related mutations in antiretroviral drug-naïve patients isolated before Initiative Congolaise d’Accès aux Antiretroviraux (ICAARV).

## Methods

Fifty blood samples from patients with evident signs of AIDS or following voluntary testing were obtained from the teaching hospital at Brazzaville (n = 48) and the regional hospitals at Pointe-Noire (n = 1) and Gamboma (n = 1) from 2002 to 2004.

HIV diagnosis, the algorithm used to confirm the HIV/AIDS infection, and DNA extractions from uncultured peripheral blood mononuclear cells (PBMCs) were previously described [9]. A nested-PCR protocol was used to obtain information on approximately 1865 bp of the *pol* gene fragment encompassing the protease and reverse transcriptase (RT) regions using the Expand Long Template PCR system (Roche) with previously described primers [10]. PCR products were purified using a Qiaquik gel extraction kit (Qiagen, Courbeouf, France), and direct sequencing was conducted with primers encompassing the *pol* region. Cycle sequencing was performed by fluorescent Dye Terminator chemistry (BDTv3.1, Applied Biosystems) per the manufacturer. Sequences were collected with an automatic genetic analyzer (3100 Avant, Applied Biosystems). Corrections and contig reconstitutions were made with SeqScape software (Applied Biosystems). Sequences were aligned under the CLUSTAL X program with the reference sequences representing the overall HIV-1 group M genetic diversity observed in Central Africa. We included all pure subtypes and the tentative subtype L variant. We also included all the sub-subtypes and reference strains for all the CRFs available in the databases and documented in Africa (CRF01, 02, 04, 05, 06, 09, 11, 13, 18, 19, 27, 28, 36, 37, and 45). Moreover, we also added other sequences of unique recombinants from DRC (MAL and NOGIL with Accession Numbers X04415 and AJ237565.1). Phylogenetic trees were generated by the neighbour-joining (NJ) method, and the reliability of the branching patterns was assessed using the bootstrap approach (confidence value for individual branch of the resulting trees evaluated with 100 bootstrap replicates). Phylogenetic analysis was conducted first for each new sequence individually. To clearly identify whether a sequence belonged to a subgroup corresponding to a CRF within a specific subtype, all HIV-1 variants, including CRFs and URFs circulating in West and Central Africa, were considered in each individual tree.

In order to confirm clusters, trees were constructed for each group of new sequences that were thought to cluster together. Finally, a general tree was made with all the sequences. As the inclusion of all sequences significantly decreased bootstrap values for certain subtypes/CRFs due to the high intra-subtype/CRF diversity; the general tree was drawn with the minimal number of references, i.e., excluding those that were not represented in this study. The clustering of each new sequence should be concordant among all the trees.

To obtain more detail about ​​recombining strains of HIV-1 circulating in the Congo, the same samples previously analyzed in the *env* region (V3–V5 loop) and gag (p24) were re-analyzed and compared to subtype designation in the *pol* gene to reflect new strains recently described.

To determine whether the viruses were recombinants, several additional analyses were performed using the Simplot version 3.5.1 software (http://www.med.jhu.edu/deptmed/sray). The percentage of similarity plots was revealed between the query group and selected groups of sequences by moving 350 nucleotides along the *pol* alignment with 20 base pair increments. The Simplot software also performed bootscanning on NJ trees using the same window and increment size and 100 replicates for each phylogeny.

The strain or sequence fragments that did not cluster with any of the known subtypes, CRFs or URFs, were submitted to a blast analysis on the Los Alamos and GenBank databases (http://www.hiv.lanl.gov/content/sequence.basis_blast/basis_blast.html) to determine whether they were related to previously described unknown fragments of other HIV-1 strains. Sequences were also analyzed for the presence of major and minor mutations in protease and reverse transcriptase genes at positions known to be associated with ARV resistance. Resistance mutations were identified using the Stanford Calibrated Population Resistance Tool [11], and samples were considered to be resistant if they contained one or more mutations as defined by the Bennett WHO list of transmitted resistance [12]. The new Congolese sequences have been deposited in EMBL for *pol* sequences, and the accession numbers are as follows: FM164884-FM164933.

## Ethical considerations

At the time of sample collection, there was no ethics committee in the country and the retrospective ethics approval was not possible to obtain. However, all of the patients were treated with the same medication. Treatments were forwarded to the doctors for additional follow-up. All patients have given their written or verbal consent in accordance with the Helsinki Declaration. No patient who did not agree to participate in the survey was discriminated. The identity of the participants was not shared throughout the process. Measures to ensure the safety of the phlebotomist and patients, such as wearing gloves, disinfection of the area to be punctured, have been ensured.

## Results

The characteristics of the studied population were as follows: the mean age was 34 years ranging from 4 to 49. In all, 64% of the samples were obtained from women, and the main route of transmission was heterosexual contact. The WHO clinical classification showed that the majority of these patients (44%) were at stage 3, followed by stage 1 (26%), stage 4 (14%) and stage 2 (10%). In all, 6% of patients had no known disease stages.

## Genetic diversity analysis

**Fig. 1:**
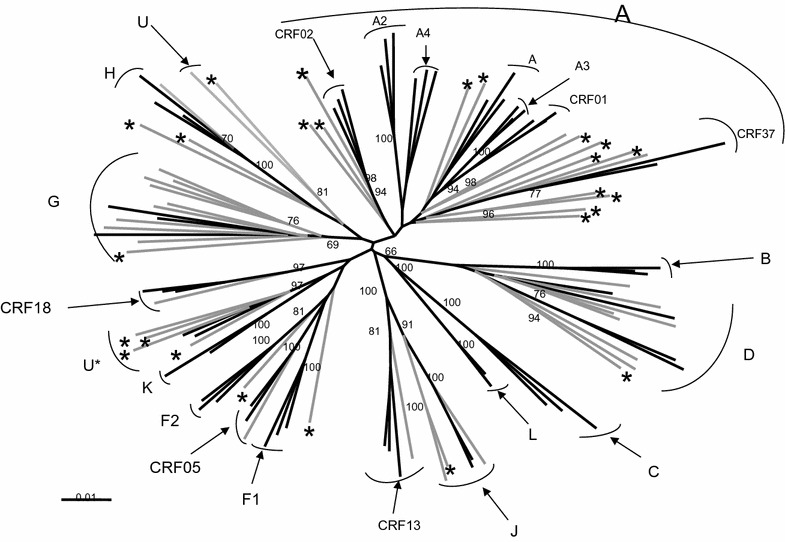
Phylogenetic relationships of 1800 unambiguously aligned nucleotide sequences representing the partial pol gene. Sequences were aligned with HIV-1 subtype/CRF reference sequences, and the phylogenetic trees were constructed with the neighbor-joining method implemented with the Clustal Program. Reference strains are in *black* and samples in *grey*. The recombined strains are indicated with an *asterisk*

Figure 1 shows the phylogenetic tree analysis of the 50 newly sequenced *pol* samples from the Congo against the reference strains for the corresponding HIV-1 variants over 1264 unambiguously aligned nucleotides, as indicated upstream. A great genetic diversity was observed, with a predominance of the A and G subtypes (n = 14, 28% and n = 10, 20%, respectively) followed by the D subtype (n = 8, 16%). A consequent number of samples (n = 9, 18%) could not be subtyped and were therefore designated U (undetermined). Among them, 8% were closely related to each other and tightly clustered with the two unique recombinants MAL and NOGIL that shared the same structure in that part of the genome (noted U*). Three samples (6%) were representative of the rare J subtype, and we found one strain (2%) of each of the subtypes F and H and of the circulating recombinants CRF05, CRF13, and CRF18, as well as CRF37.

**Fig. 2:**
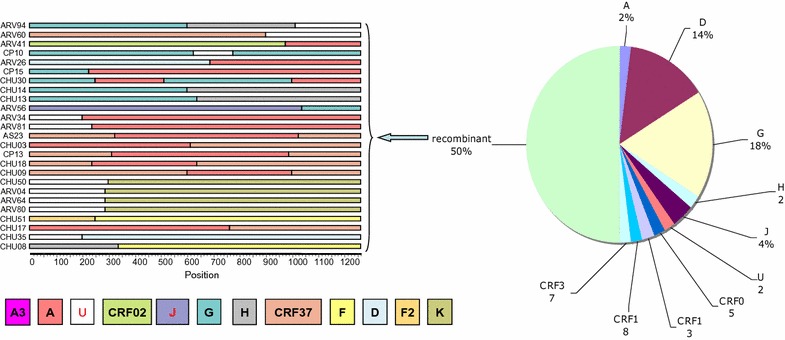
Schematic representation of the mosaic pol sequences. The bootstrap values supporting the subtype assignments in the subregion trees were all above 80%

Each of the new strains was submitted in Simplot and subjected to bootscan analysis as previously described. The main result was that 50% of the new strains were recombinants in *pol*, as indicated with an asterisk at the top of each branch. Figure 2 illustrates in detail the structure of the mosaic sequences and the definitive distribution of the *pol* HIV-1 variants in Congo. In fact, the major changes in the range of recombinants were caused by the A subtype samples and the undetermined strains. Among 14 subtypes of A sequences, only one was a wild-type A subtype. Other strains were recombinants with unknown fragments or fragments linked to A3, G, CRF02 or CRF37 HIV-1 variants. Interestingly, we found that four new strains shared the same structure (CRF37/A/CRF37), and two of these sequences had the same recombination breakpoints. As a consequence, the real prevalence of A subtypes in this study decreased from 28 to 2%. From the undetermined Congolese strains, one (subtype D in *gag* and *env*) seemed to be recombinant, and another involved three different variant fragments (G/H/U). All other undetermined sequences were constituted with two (02) different HIV-1 subtypes (one H/F, two identical G/H, and four identical U/K corresponding to the mosaic structure of the old MAL and NOGIL URFs). The ten G and eight D subtype congolese strains each accounted for one more recombinant strain (G/U/G and U/D, respectively), and one of the three J subtype strains was a mosaic J/G.

Unidentified strains were submitted to a blast analysis, as indicated earlier, to determine whether they may be linked to other previously published HIV-1 strains. We found that the four strains clustering together with MAL and NOGIL viruses (U*) have several other representatives in the DRC, (97CD.MBFE185, 97CD.MBS3010, 02CD.KP061, 02CD.KP0976) and in Senegal (98SN.40HALD) [10]. A neighbour-joining tree of the same *pol* fragment expressing protease and RT revealed that all these strains clustered together with 100% bootstrap. Subsequent simplot and bootscan analyses confirmed that they shared the same recombinant U/K structure in that fragment. The recombinant G strain from Congo clustered with 100% bootstrap supports 2 previously published strains from the DRC 97CD.EQS39 [10] and 02CD.KS108 [6] inside the G subtype branch. Again, subsequent simplot and bootscan analyses revealed the same mosaic G/U/G structure for the 3 strains with similar breakpoints locations. Other undetermined strains of Congo, particularly the mosaic CRF37/A/CRF37 viruses, were also tested against comparable recombinant strains found in DRC and Cameroon. We did not observe any clear clustering or similar breakpoints; however, the structure of these strains involved the same subtypes/CRFs.

The majority of these new Congolese strains had also been previously sequenced in the p24 region of the *gag* gene and/or in the V3–V5 region of the *env* gene, as published by Niama et al. [9]. When this study was conducted, several HIV-1 variants were not yet described, and we have analyzed again all sequences, including more recently described subtypes and CRFs. The subtype distribution was globally confirmed and remained dominated by the subtypes G, A and D (Table 1). However, previously, subtyped A strains strongly clustered with the CRF37 reference in the *gag* region confirmed that these strains were circulating in central Africa and in the country. In the present study, among 50 analyzed samples, only two subtype G and one subtype D strains were not sequenced in either *gag* or *env*. Six samples were subtyped in one of the regions, and 41 samples were available in *gag, pol,* and *env*. Eight strains that were non mosaïc in *pol* did not display the same subtype/CRF in the other sequenced regions, leading to a global prevalence of 66% recombinant strains. The CRF13 strain was found to be of subtype A in *env*; the CRF37 in *pol* and *gag* was CRF19 in *env*; two G subtypes in *gag* and *pol* were either A or CRF06 in *env*; one G subtype in *pol* was J in *gag* and *env*; the two J subtypes in *pol* involved either D, H or G in the other regions; and the non-mosaic undetermined strain was subtype D in *gag* and *env*. Interestingly, 3 of the 4 *pol* recombinants CRF37/A/CRF37 were CRF37 in *gag* and A in *env*, and 2 of the 4 undetermined U* *pol* sequences were forming a specific A cluster in *gag* p24 and in the V3–V5 *env* regions. These findings suggested that the majority of HIV-1 strains circulating in the Republic of Congo display a complex mosaic structure.

**Table 1:** Genetic subtypes in the gag p24 env V3–V5 and pol (Pr and RT) regions

gag subtype	N	env subtype	N	*pol* subtype	N
A	1	A	1	A	1
A	1	A	1	A3/A	1
A	1	A	1	G/A/G/A	1
A	1	A	1	U/K	1
A	1	A	1	CRF37/A/CRF37	1
A	1	CRF01	1	U/A	1
A	1	J	1	CRF37/U (GJ)	1
A3	1	CRF06	1	CRF02/A	1
CRF05	1	F1	1	CRF05	1
CRF18	1	A	1	A/CRF37	1
CRF18	1	CRF18	1	CRF18	1
CRF18	1	CRF18	1	G/A	1
CRF18	1	U	1	G/H/U	1
CRF37	1	A	1	CRF37/A/CRF37	2
CRF37	1	CRF19	1	CRF37	1
CRF37	1	U	1	CRF37/A/CRF37	1
D (D and D’)	5	D	6	D	4
D (D and D’)	1	D	1	U	1
D (D and D’)	1	D	1	U/D	1
D (D and D’)	1	U	1	D	1
F2	1	ND	1	F2/F (F not F1, not F2)	1
G	1	A	1	G	1
G	1	CRF06	1	G	1
G	1	G	1	G	3
G	1	U	1	G/H	1
G	1	H	1	G/H	1
G	1	ND	1	G/U/G	1
G	1	ND	1	J	1
G	1	ND	1	A/CRF37	1
G	1	ND	1	G	1
G	1	ND	1	U/K	1
H	1	H	1	H	1
J	1	J	1	G	1
J	1	U	1	J/G	1
J	1	ND	1	G	1
ND	3	A	1	CRF13	1
ND	1	C	1	U/A	1
ND	1	D	1	J	1
ND	1	ND	2	D	1
ND	1	ND	1	G	1
ND	1	ND	1	G/U/G	1
U	1	F1	1	H/F	1
U	1	A	1	U/K	1
U*	1	A	1	U/K	1

*ND* not done, *n* number of sequences

* Strains clustering with MAL and NOGIL viruses

## Antiretroviral resistance

Analysis of the *protease* (*Prot*) gene showed no major mutations associated with the resistance to *Protease* Inhibitor resistance. However, polymorphisms, which were naturally occurring amino acids changing at sites associated with resistance, were also observed, and all samples carried more than 5 of these secondary mutations. The most frequent were scored at positions M36 (90%), H69 (82%), I13 (80%), K20 (48%), L63 (46%), E35 (44%), V82 (28%), I62 (24%), L10 (20%), and V77 (12%). Regarding the reverse transcriptase segment, the following resistance-related mutation, M230L (n = 1, 2%), which is associated with a high level of resistance to delavirdine and nevirapine and causes an intermediate resistance to efavirenz and etravirine, was observed in one sample from Pointe-noire (CgAS23) involving the genomic complex structure gagCRF37/envA/polCRF37/A/CRF37. In addition, one sample (CgCHU38) from a patient attending the Centre Hospitalier et Universitaire de Brazzaville (CHU-B) and subtyped as G in all 3 regions has the mutations Y181C, M41L and T215Y. Other minor mutations were also found, and the most prevalent were at positions V35 (98%), D177 (76%), V60 (62%), Q174 (58%), D123 (50%), T39 (48%), V135 (34%), K173 (33%), K13R (26%), D121 (24%) and other less prevalent mutations.

## Discussion

The present study aimed to determine the prevalence of strains resistant to anti-retroviral drugs in untreated HIV-1 infected patients selected in the Republic of Congo and to contribute to the HIV-1 genetic diversity. Phylogenetic analyses of protease and *RT* in the *pol* gene confirmed the strain distributions previously observed in the *gag* (p24) and *env* (v3–v5) regions [9]. Surprisingly, the subtype A, which was one of the most important HIV-1 circulating subtypes in Congo (56.25% in 2004 [7] and 36.5% in *gag* in 2006 [8]), represents now only 2% (1 strain). This weak proportion can be explained by the fact that the majority of strains (13 of 14) classified as A in the pol gene were revealed to be recombinants in the bootscan analysis. This tendency for the recombination of subtype A has been highlighted in previous studies in 2006. It was reported that one of these three subtypes, found as A in *gag*, were recombinants in the *env* regions [9]. In neighbouring countries such as DRC, it was found that 70% of strains that classified as A in *env* (V3–V5) displayed different subtypes in *pol*. In Cameroon, the recombinant CFR02_AG located in “group” A were predominant [13].

Another important feature of the genetic diversity in Congo was the high proportion of strains that could not be classified into the current subtypes/CRFs and the extremely high frequency of recombinant strains (66%). This proportion appears to be higher than those previously reported in the country: in 2004 and 2006 [8, 9], it was found that 27 and 20% of the virus, respectively, displayed different subtype/CRF designations. In DRC, the proportion of recombinant strains in the *pol*/*env* genomic region was 42% in 2002 [6]. In Angola, 47.1% of strains were recombinant [14]. The importance of recombination events may depend on the number of studied regions. In the present study, three different regions were available, and the conditions for detecting more recombination were present.

This study provides additional knowledge of the baseline pattern of HIV-1 transmission of strains harboring the major drug resistance mutations just before the large-scale implementation of ICAARV. No major resistance associated to the protease inhibitors was found. However, many polymorphisms were noted. The majority of our population was at stage 3 or 4 according to the WHO classification. The advanced AIDS stage could explain the absence of major mutations associated with resistance to the anti-protease drug according to the previous study of Wensing et al. [15]. An important polymorphism of the protease gene was also observed that could affect the viral fitness or predict the viruses to be of potentially greater risk of virological failure during protease inhibitor (PI) therapy [16]. For the RT genomic region, the mutations Y215C and M230N were observed at Brazzaville and Pointe-noire, respectively. These mutations are strongly associated with a high level of resistance to the INNRTI. One sample obtained at Hopital Adolphe Cissé de Pointe-Noire (gagCRF37/envA/polCRF37/A/CRF37) was female, but previous history of nevirapine drug use, i.e., through the Mother to Children Transmitted program (MTCT), was not documented. Additionally, the sample harboring the mutation T215Y and the TAMs M41L and Y181C sampled at the CHU de Brazzaville (subtype G) was male, but the transmission of these HIV-1 mutations from nevirapine-exposed patients can be suspected.

Finally, the global prevalence of transmission of drug resistant HIV-1 strains in our study can be estimated as 4%. However, according to Palma et al. [17] this prevalence might be underestimated. Indeed, it is hypothesized that in the absence of drug selective pressure, some transmitted drug-resistance mutations may revert to wild type. The prevalence of drug resistance in Congo is lower than those generally reported in Europe where subtype B dominates. Indeed, in Portugal [17] in 2007, Palma AC et al. found that 7.78% of the newly diagnosed patients were infected by the strains harboring the mutations conferring resistance to one or three drug classes. In Greece, 9% of resistant HIV-1 strains were found in the primary diagnosis patients [18]. Similar studies conducted in Bulgaria by Santoro et al. [19], and in Luxemburg by Schmit in 2000 [20] showed that 9.1 and 13% of strains, respectively, were naturally resistant to certain anti-retroviral molecules. Our data do not differ significantly from those reported by Kousiappa in 2009 [21] and by Juhász E in 2008 [22] where 2.7 and 3.5%, respectively, of resistance to HIV-1 drugs were found in Cyprus and Hungary. In Asia, the lowest prevalence of transmitted drugs was reported. Indeed, in recent studies conducted in Cambodia and Vietnam, 1.49 and 2.6% of resistant strains were reported, respectively [23, 24]. In South Korea, the prevalence of drug-resistant HIV-1 in ART-naive patients was estimated at 12% in a recent study [25]. The studies conducted in Africa with samples obtained from persons not experiencing the HAART treatment showed globally a weak prevalence of the wild strains harboring such mutations. Studies in Ethiopia [26], Burundi [27], Swaziland [28], Kenya [29] and Rwanda [30] showed prevalence of 2.2, 0.5, <5%, 3.2 and 3.6%, respectively. In Mozambique, the rate of transmitted drug was lower in Maputo, the capital city (<5%) and more than 5% in Beira [31]. A high level of drug resistance was reported in Mozambique [31]. In neighbouring countries like Angola, a study conducted in different areas of the country revealed “an unexpected high frequency of DRM to RT inhibitors (16.3%) in patients who have reported no antiretroviral usage.”

## Conclusion

In the present work, we showed that the high genetic diversity of HIV-1 strains isolated in the Republic of Congo was confirmed, and the majority of these harbored a complex genomic structure. The prevalence of drug resistance cases of 4% might presage the eventual spread of drug-resistant HIV in the general population. Thus, continuous monitoring of treatment-naïve HIV-1-infected populations 15 years after the implementation of the governmental initiative to provide therapy should be considered.
